# Low immunogenicity of mouse induced pluripotent stem cell-derived neural stem/progenitor cells

**DOI:** 10.1038/s41598-017-13522-w

**Published:** 2017-10-11

**Authors:** Go Itakura, Masahiro Ozaki, Narihito Nagoshi, Soya Kawabata, Yuichiro Nishiyama, Keiko Sugai, Tsuyoshi Iida, Rei Kashiwagi, Toshiki Ookubo, Kaori Yastake, Kohei Matsubayashi, Jun Kohyama, Akio Iwanami, Morio Matsumoto, Masaya Nakamura, Hideyuki Okano

**Affiliations:** 1 Department of Physiology, Keio University School of Medicine, 35 Shinanomachi, Shinjuku, Tokyo, 160-8582 Japan; 2 Department of Orthopaedic Surgery, Keio University School of Medicine, 35 Shinanomachi, Shinjuku, Tokyo, 160-8582 Japan

## Abstract

Resolving the immunogenicity of cells derived from induced pluripotent stem cells (iPSCs) remains an important challenge for cell transplant strategies that use banked allogeneic cells. Thus, we evaluated the immunogenicity of mouse fetal neural stem/progenitor cells (fetus-NSPCs) and iPSC-derived neural stem/progenitor cells (iPSC-NSPCs) both *in vitro* and *in vivo*. Flow cytometry revealed the low expression of immunological surface antigens, and these cells survived in all mice when transplanted syngeneically into subcutaneous tissue and the spinal cord. In contrast, an allogeneic transplantation into subcutaneous tissue was rejected in all mice, and allogeneic cells transplanted into intact and injured spinal cords survived for 3 months in approximately 20% of mice. In addition, cell survival was increased after co-treatment with an immunosuppressive agent. Thus, the immunogenicity and post-transplantation immunological dynamics of iPSC-NSPCs resemble those of fetus-NSPCs.

## Introduction

The development of a method for generating induced pluripotent stem cells (iPSCs) has greatly enhanced stem cell transplantation therapeutics^1–3^. Our group has previously reported the efficacy of neural stem/progenitor cells derived from human and rodent iPSCs (iPSC-NSPCs) transplanted in animal models of spinal cord injury^4–7^. An advantage of iPSCs is the feasibility of autologous transplantation. However, several major limitations affect their clinical use: the long time (approximately 6 months) required to induce iPSC differentiation into mature cell types of interest; extremely high costs attributable to a poor economies of scale; and the need to validate the safety and efficacy of individual iPSC lines^8–10^. Therefore, allogeneic transplantation combined with iPSC banks is a more viable strategy.

However, allograft transplantations are associated with immune rejection. The immunogenicity of fetal neural stem/progenitor cells (fetus-NSPCs)^11–15^ and embryonic stem (ES) cells^16,17^ is well-documented, whereas no consensus has been reached on the immunogenicity of iPSCs^18,19^. iPSC-derivatives exhibit low immunogenicity *in vitro* ^20^, but a paucity of studies have evaluated transplanted iPSC-derived cells to assess the presence or absence of immune rejections *in vivo*.

Zhao *et al*. speculated that iPSCs are more immunogenic than ES cells and may be subject to immune rejection even under syngeneic conditions^21^, but these findings were based on the immune response to undifferentiated iPSCs and teratomas differentiated from iPSCs^22^. In contrast, studies on the immune response to transplanted iPSC-derivatives showed an absent immune rejection in syngeneic settings^23,24^; however, the immunogenicity of allogeneic iPSC-derived cells has yet to be studied extensively in diverse tissue sites. Previous studies showed that immune rejection occurs in all cases of allogeneic transplantation of iPSC-derived cells into subcutaneous tissue, bone marrow^24^, and myocardium^25^. Notably, the immunodynamics of these allografts in the spinal cord remain unclear. Here, we investigated the *in vitro* and *in vivo* immunogenicity of mouse iPSC (miPSC)-NSPCs in syngeneic and allogeneic settings and then compared these results with those obtained for fetus-NSPCs.

## Results

### Characterization of lentivirally transduced mouse fetus-NSPCs and miPSC-NSPCs

**Figure 1:**
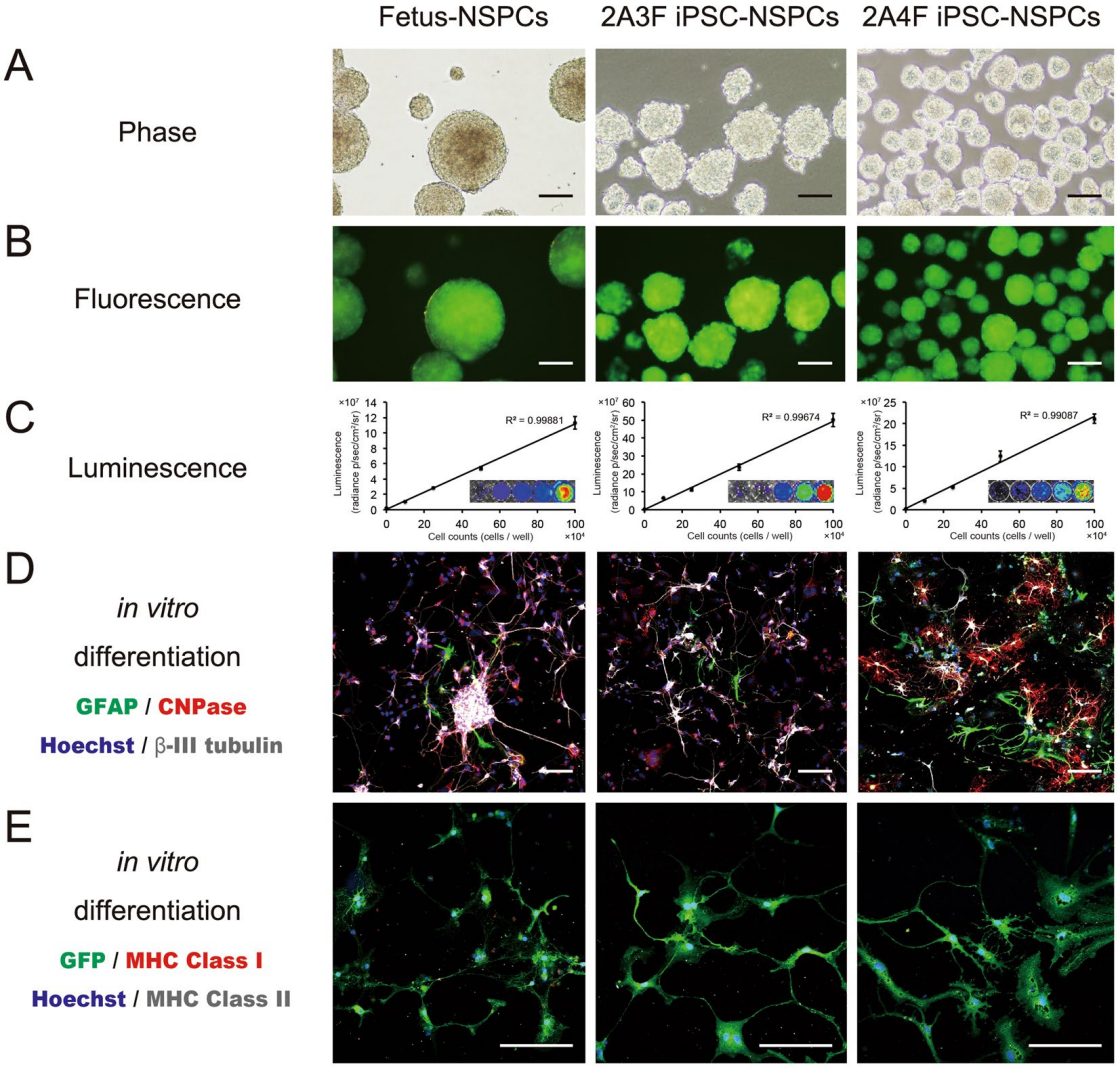
Characterization of lentivirally transfected mouse fetus-NSPCs and mouse iPSC-NSPCs *in vitro*. (**A**) Phase contrast, (**B**) fluorescence, and (**C**) luminescence images. (**D**,**E**) Immunostained images of neurospheres derived from mouse fetus-NSPCs and miPSC-NSPCs (after *in vitro* differentiation with antibodies against glial fibrillary acidic protein (GFAP), CNPase, βIII tubulin, GFP, MHC class I, MHC class II, and Hoechst). Bioluminescence imaging was conducted in various numbers of fetus-NSPCs and iPSC-NSPCs (0, 1 × 10^5^, 2.5 × 10^5^, 5 × 10^5^, and 1 × 10^6^ cells per well). A correlation between luminescence and the number of cells was confirmed. Fetus-NSPCs and iPSC-NSPCs differentiated into β-III tubulin + neurons, GFAP + astrocytes, and CNPase + oligodendrocytes *in vitro* (**D**). The expression of MHC class I or II was not observed (**E**). Scale bars = 1,000 µm in (**A**) and 100 µm in (**B**), (**D**), and (**E**).

Fetus-NSPCs from transgenic *ff*Luc-cp156 mice, which express a yellow variant of Aequorea GFP and firefly luciferase^26^, and two lines (2A3F and 2A4F) of iPSC-NSPCs were used for our *in vitro* studies. The *ffLuc* construct was lentivirally transduced into the iPSC-NSPCs to facilitate fluorescence and luminescence observations, and a correlation between luminescence and cell number was confirmed (Fig. 1A–C).

After inducing terminal differentiation, both fetus-NSPCs and iPSC-NSPCs differentiated into Tuj-1 + neurons, glial fibrillary acidic protein (GFAP) + astrocytes, and CNPase + oligodendrocytes (Figs 1D and S1). These terminally differentiated cells did not express MHC (Fig. 1E).

### Fetus-NSPCs and iPSC-NSPCs show a similarly low expression level of immune-related proteins

**Figure 2:**
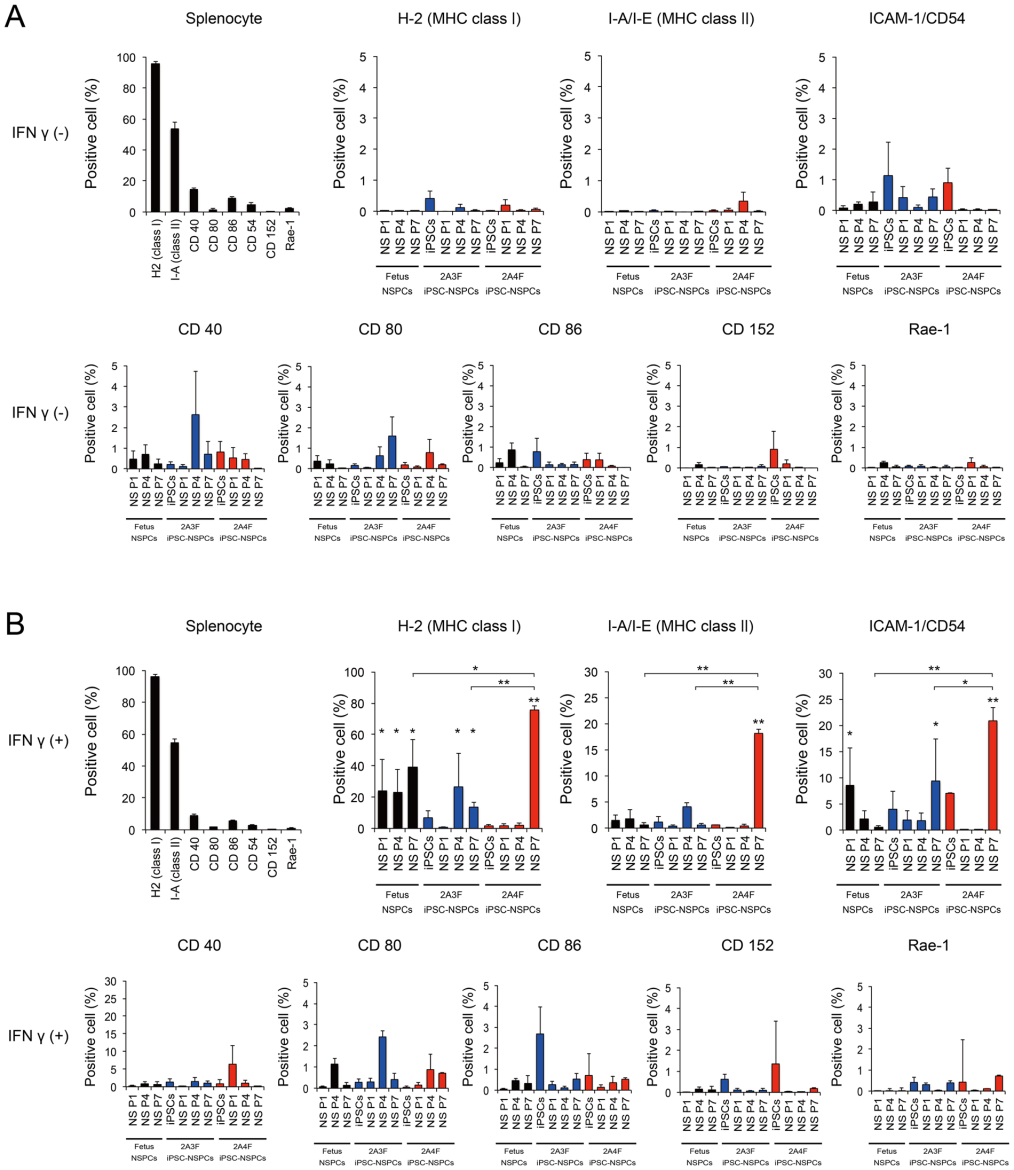
Fetus-NSPCs and iPSC-NSPCs showed a similar low expression level of immune-related proteins. The expression levels of MHC class I (H-2), MHC class II (I-A), ICAM-1 (CD54), co-stimulatory molecules (CD40, CD80, and CD86), CTLA4 (CD152), and NKG2DL (Rae-1) were assessed using flow cytometry in mouse spleen cells, fetus-NSPCs, iPSCs, and iPSC-NSPCs with (**A**) or without (**B**) the addition of IFNγ (n = 3 independent experiments). Values are shown as the mean ± SEM. *P < 0.05 and **P < 0.005, one-way ANOVA followed by the Tukey–Kramer test.

Using flow cytometory, the immunological expressions of surface antigen markers, including MHC class I and II molecules, leukocyte adhesion molecule CD54, co-stimulatory molecules CD40, CD80, and CD86, CD152 (cytotoxic T lymphocyte antigen 4; CTLA4), and NKG2DL (Rae-1), were analyzed in fetus-NSPCs at passages 1 (P1), 4 (P4), and 7 (P7), the iPSCs from which the 2A3F and 2A4F lines were derived, iPSC-NSPCs at P1, P4, and P7, and mouse spleen cells (positive control). In a normal culture environment, the expression levels of these immunological surface antigen markers were less than 5% in fetus-NSPCs, iPSCs, and iPSC-NSPCs. In addition, no significant differences were seen according to passage number or between fetus-NSPCs and iPSC-NSPCs, and both cell populations scarcely expressed these surface antigens (Fig. 2A). In the presence of the pro-inflammatory cytokine interferon γ (IFNγ), the expression levels of MHC class I and II and CD54 markedly increased (Fig. 2B). In 2A4F iPSC-NSPCs P7, expression of these markers increased significantly compared with fetus-NSPCs and 2A3F iPSC-NSPCs P7. However, in the other samples, the marker expression profiles and levels were similar between iPSC-NSPCs and fetus-NSPCs (Fig. 2B). These results suggest that external factors, such as pro-inflammatory cytokines like IFNγ, significantly influence iPSC-NSPC immunogenicity. Under normal conditions, however, the expression of immunological surface antigen markers was very low in both iPSC-NSPCs and fetus-NSPCs. Increased expression levels in iPSC-NSPCs and in fetus-NSPCs in response to immunogenic factors nonetheless remained very low compared with levels in spleen cells.

### Fetus-NSPCs and iPSC-NSPCs triggered allogeneic peripheral blood mononuclear cell (PBMC), but not syngeneic PBMC, proliferation *in vitro*

**Figure 3:**
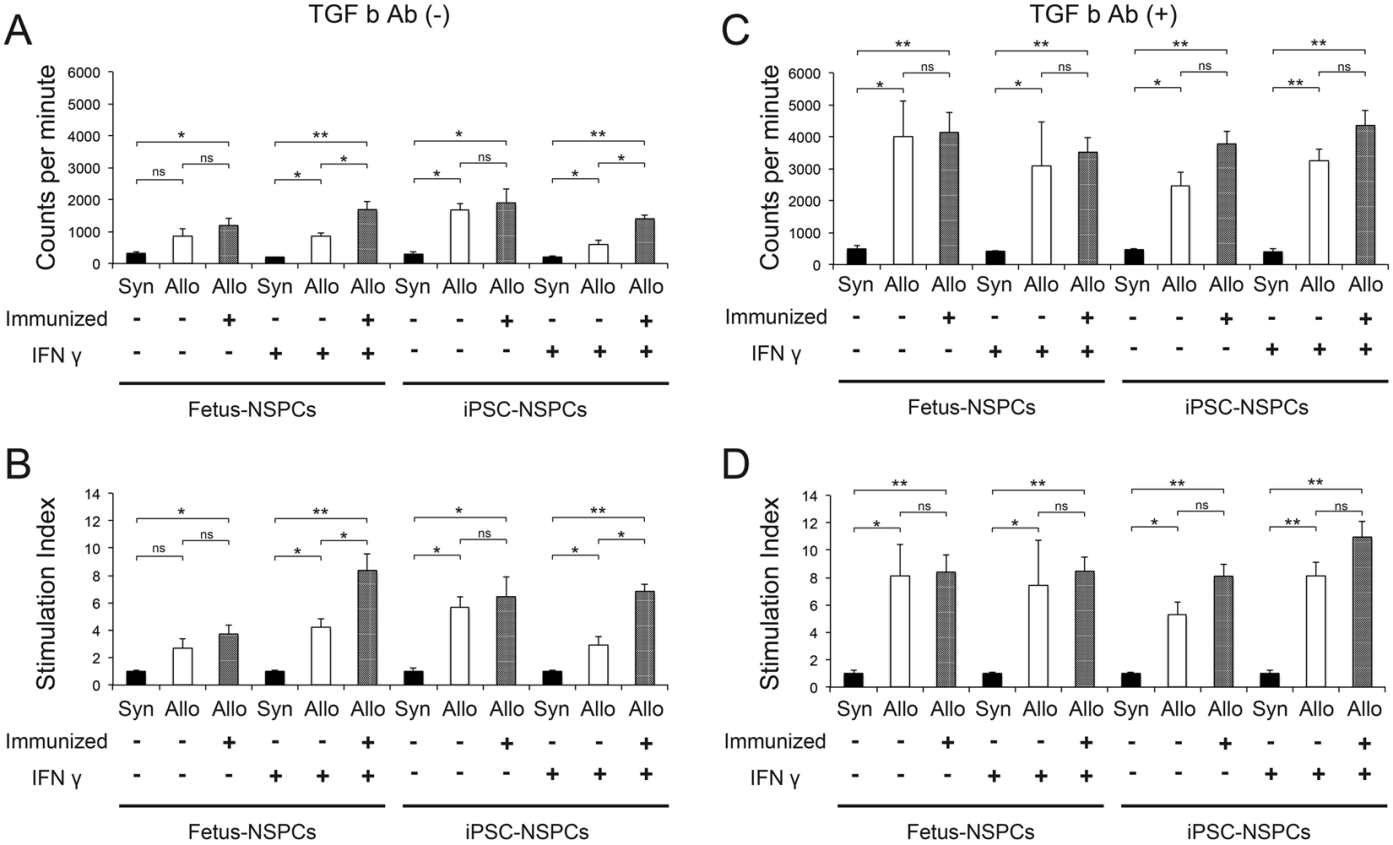
Fetus-NSPCs and 2A4F iPSC-NSPCs triggered allogeneic PBMC, but not syngeneic PBMC, proliferation *in vitro*. Fetus-NSPCs and 2A4F iPSC-NSPCs were irradiated and co-cultured with syngeneic mouse (C57BL6/J) PBMCs and allogeneic mouse (BALB/cA) PBMCs (pre-immunized and post-immunized) at a ratio of 1:10 with or without IFNγ and anti-TGFβ receptor antibodies for 5 days (n = 3 independent experiments). (**A**,**C**) CPM measured in MLR assays without (**A**) or with (**C**) the anti-TGFβ antibody. (**B**,**D**) The SI was calculated as the ratio of the CPM in each MLR assay relative to the CPM in a syngeneic MLR assay. Values are shown as the mean ± SEM. *P < 0.05 and **P < 0.005, unpaired two-tailed Student’s t-test or one-way ANOVA followed by the Tukey–Kramer test. ns = not significant.

To assess the *in vitro* response of lymphocytes to NSPCs, C57BL6/J mouse lymphocytes (syngeneic) or BALB/cA mouse lymphocytes (allogeneic, immunized [−]: intact mice; immunized [+]: mice that previously rejected NSPCs transplanted into the spinal cord) were co-cultured with fetus-NSPCs or 2A4F iPSC-NSPCs at P4 and used in mixed lymphocyte reaction (MLR) assays^14,27,28^. In fetus-NSPCs and 2A4F iPSC-NSPCs, the counts per minute (CPM) were higher for allogeneic cells than syngeneic cells under all experimental conditions. IFNγ increased the CPM and stimulation index (SI) for allogeneic fetus-NSPCs, but not iPSC-NSPCs, when compared with normal conditions. In immunized (+) lymphocytes, IFNγ increased the CPM and SI, indicating an increase in lymphocyte activity. However, no significant differences were observed under normal conditions (Fig. 3A,B).

Previous studies have reported that transforming growth factor β(TGFβ) exerts an immunosuppressive effect in NSPCs^27,29^; thus, we investigated the effects of treatment with a TGFβ-neutralizing antibody^30^. The CPM and SI significantly increased in fetus-NSPCs and 2A4F iPSC-NSPCs after treatment (Fig. 3C,D), indicating that TGFβ-dependent immunoregulation occurs in mixed cultures of lymphocytes and NSPCs.

### Survival of grafted fetus-NSPCs and iPSC-NSPCs in syngeneic and allogenic settings

**Figure 4:**
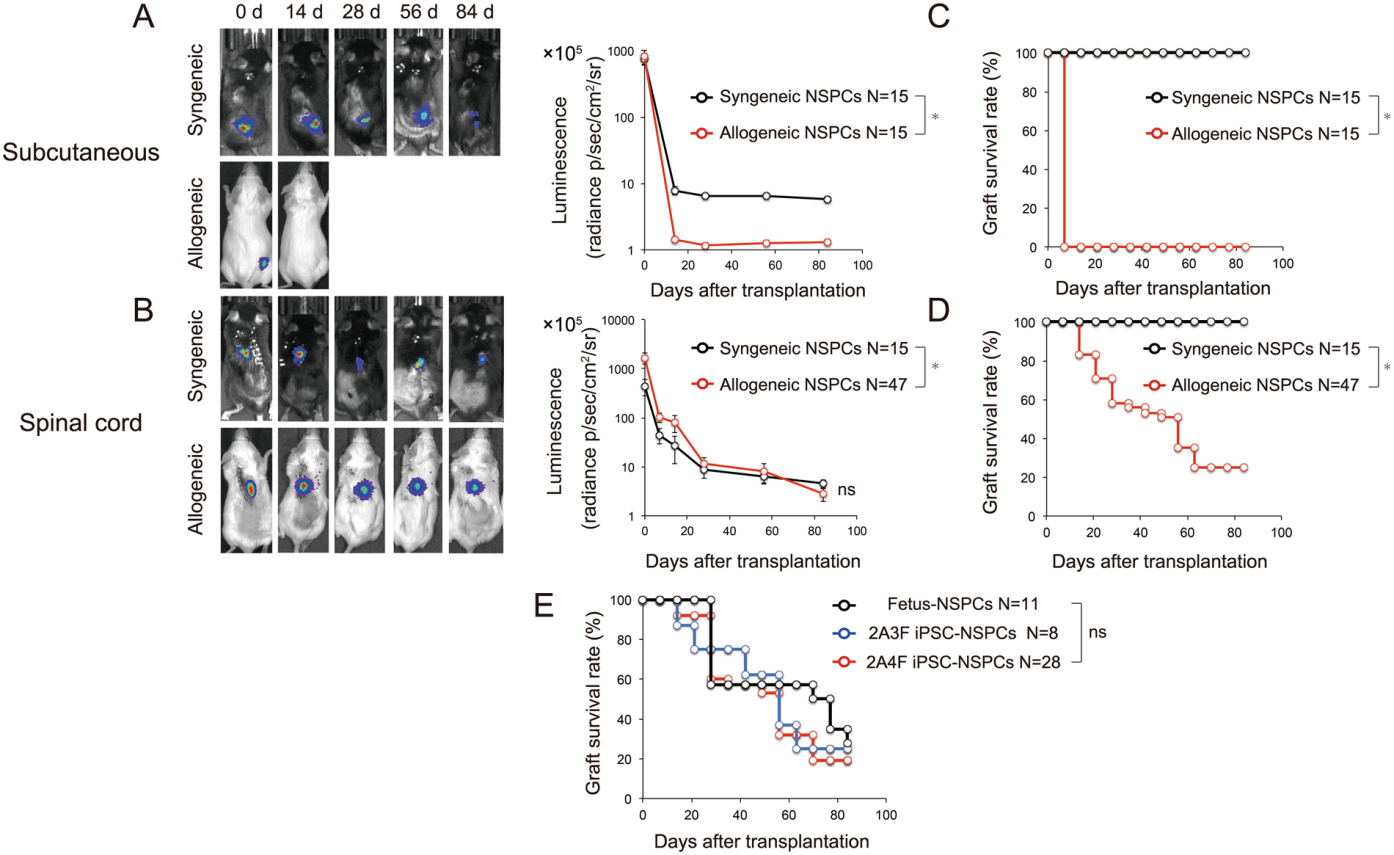
Bioluminescence and graft survival rate after transplantation of fetus-NSPCs and iPSC-NSPCs into syngeneic and allogeneic mice. (**A**,**B**) Bioluminescence images of representative mice at 0, 14, 28, 56, and 84 days after transplantation, and quantitative analyses of CPM derived from grafted fetus-NSPCs and two lines of iPSC-NSPCs (2A3F and 2A4F) in subcutaneous lesions and spinal cords (black line = syngeneic transplantation; red line = allogeneic transplantation). (**C**,**D**,**E**) Graft survival rates after transplantation into subcutaneous lesions (**C**) and spinal cords (**D** and **E**). The black line shows the fetus-NSPCs transplantation group, the blue line shows the 2A3F iPSC-NSPCs transplantation group, and the red line shows the 2A4F iPSC-NSPCs transplantation group. Values represent the mean ± SEM. *P < 0.05, one-way ANOVA followed by the Tukey–Kramer test. The graft survival rates were assessed using Kaplan–Meier survival curves. *P < 0.05, Gehan-Breslow-Wilcoxon test. ns, not significant. In (**A**–**E**), N indicates the number of mice.

Fetus-NSPCs and two iPSC-NSPCs lines were transplanted into subcutaneous tissue (Fig. 4A, black line) and intact spinal cord (Fig. 4B, black line) of MHC-matched (syngeneic) C57BL6/J mice. Fetus-NSPCs and iPSC-NSPCs survived in subcutaneous tissue and spinal cord for 12 weeks after syngeneic transplantation in all mice (subcutaneous: fetus-NSPCs, n = 5/5; 3 F iPSC-NSPCs, n = 5/5; 4 F iPSC-NSPCs, n = 5/5; spinal cord: fetus-NSPCs, n = 5/5; 3 F iPSC-NSPCs, n = 5/5; 4 F iPSC-NSPCs, n = 5/5) (Fig. 4C,D). These results are inconsistent with a previous study that showed the subcutaneous transplantation of iPSCs in syngeneic mice induced immune responses and caused regression of iPSC-derived teratomas in some mice^21^.

Next, we transplanted the same cell lines into the subcutaneous tissue (Fig. 4A, red line) and intact spinal cord (Fig. 4B, red line) of completely MHC-mismatched (allogeneic) BALB/cA mice. Immune rejection occurred and the transplanted cells disappeared from the subcutaneous tissue of all mice within 2 weeks (fetus-NSPCs, n = 0/5; 3F iPSC-NSPCs, n = 0/5; 4 F iPSC-NSPCs, n = 0/5) (Fig. 4C), which is consistent with previous results^24^. Immune responses were also induced during intraspinal transplantation. However, transplanted cells survived for 12 weeks after transplantation in approximately 20% of the mice despite the mismatch of MHC class I and II (fetus-NSPCs, n = 3/11; 3 F iPSC-NSPCs, n = 2/8; 4 F iPSC-NSPCs, n = 4/26) (Fig. 4D). No significant differences in graft survival rates were found among groups transplanted with fetus-NSPCs and 2A3F and 2A4F iPSC-NSPCs (Fig. 4E).

### Inflammatory cells are recruited to surrounding grafts even in immunologically privileged sites, such as spinal cord

**Figure 5:**
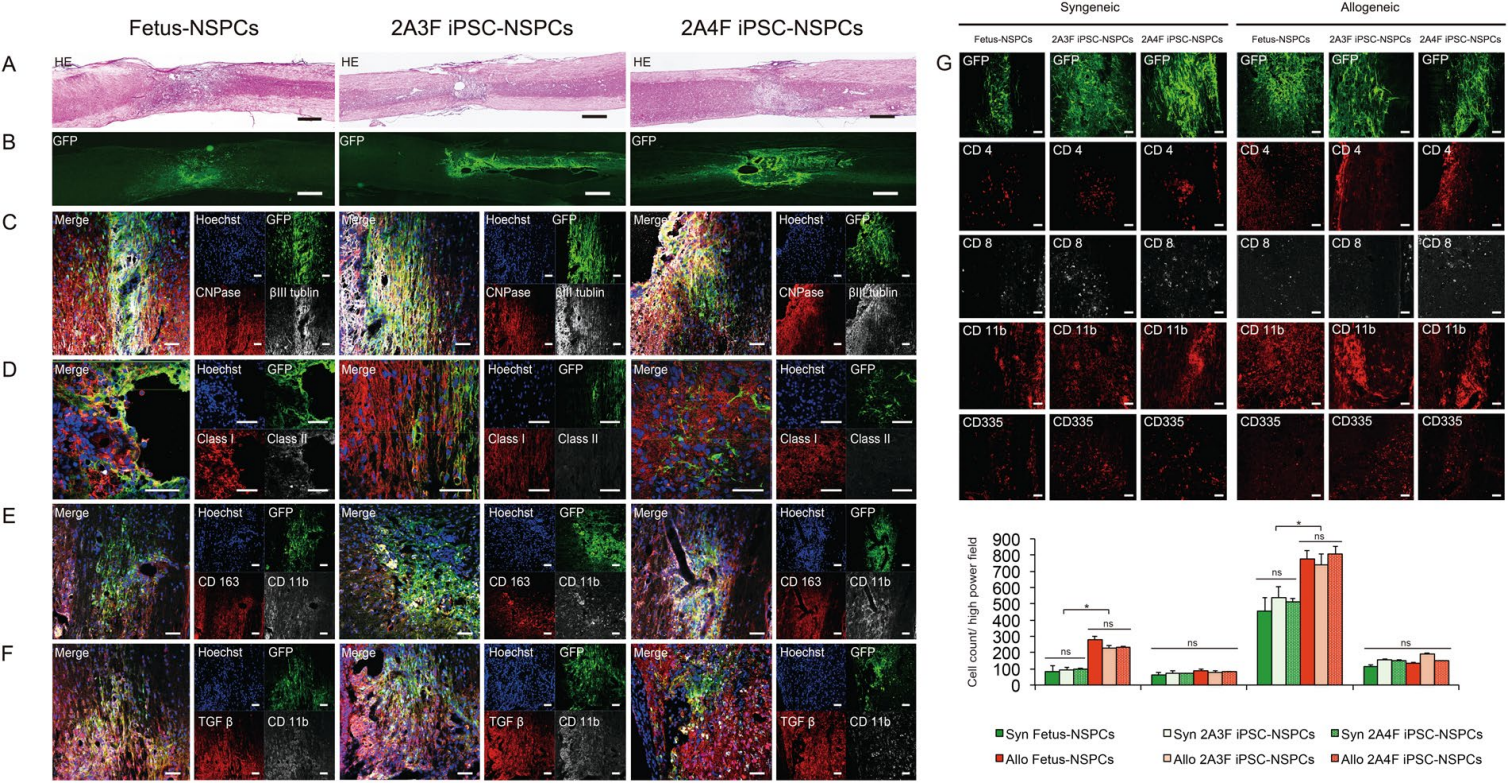
Histological examination of grafted syngeneic and MHC-mismatched allogeneic mice spinal cords. Fetus-NSPCs and iPSC-NSPCs (2A3F and 2A4F) transplanted into allogeneic hosts were stained with hematoxylin-eosin (**A**) and immunostained with antibodies against GFP, βIII tubulin, CNPase, MHC class I, MHC class II, CD163, TGFβ and Hoechst (**B**–**F**). All cell populations differentiated into three neural lineages. MHC expression was observed in a very small proportion of these cells after transplantation. Inflammatory cell infiltration was more obvious in allogeneic transplantation than syngeneic transplantation. Many of the infiltrating CD11b + macrophages were positive for CD163 and TGFβ. (**G**) GFP, CD4, CD8, CD11b, and CD335 staining and quantified of inflammatory cells in mice with surviving syngeneic or allogeneic grafts. CD4 + and CD11 + cells infiltrated the allogeneic host spinal cord significantly more than syngeneic host spinal cord. Values are shown as the mean ± SEM. *P < 0.05, unpaired two-tailed Student’s t-test and one-way ANOVA followed by the Tukey–Kramer test. ns, not significant. Scale bars = 500 µm in (**A**) and (**B**) and 100 µm in (**C**–**G**).

NSPCs were histologically examined at 3 months after syngeneic and allogeneic intraspinal transplantation. Consistent with our bioimaging results, green fluorescent protein (GFP) + transplanted cells were present in all syngeneic mice and some allogeneic mice (Fig. 5B). Differentiation into βIII tubulin + neurons and CNPase + oligodendrocytes was observed in all groups (Fig. 5C). A previous study found that NSPCs expressed MHC after differentiation^12^, We found that few transplanted cells expressed MHC class I and II, whereas inflammatory cells that infiltrated the grafts clearly expressed these markers (Fig. 5D).

A small population of transplanted cells expressed MHC, which implies that CD335 + (natural killer [NK] cell marker) NK cells and other inflammatory cells accumulated in grafts as an immune response to transplanted cells. Numerous infiltrating macrophages were identified as CD163 + M2 macrophages (Fig. 5E), and although very few transplanted cells were TGFβ + , many cells surrounding the grafts were TGFβ + (Fig. 5F). We assessed the infiltration of inflammatory cells into the spinal cord in syngeneic and allogeneic settings by staining for CD4, CD8, CD11b, and CD335 (Fig. 5G). Inflammatory cells infiltrated grafts, including those in the spinal cord; however, the infiltration of inflammatory cells was more pronounced in allogeneic grafts than syngeneic grafts, suggesting that the immune response remained active at 12 weeks after transplantation (Fig. 5G).

### Survival of fetus-NSPCs and iPSC-NSPCs grafted into intact and injured spinal cords

**Figure 6:**
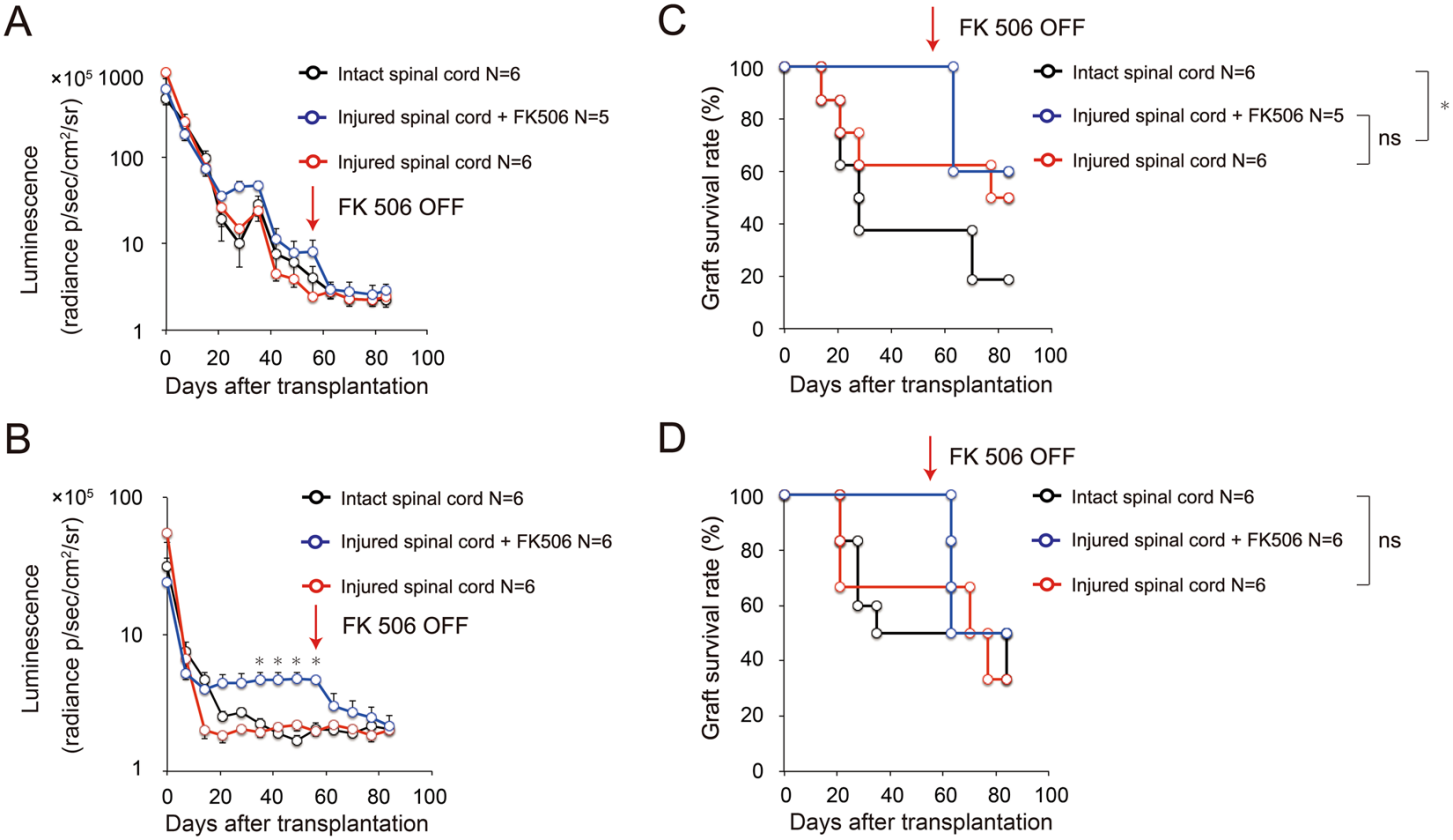
Bioimaging and graft survival rate after transplantation of fetus-NSPCs and iPSC-NSPCs into intact and injured spinal cords (with or without FK506) in MHC-mismatched BALB/cA mice. Quantitative analyses of the CPM derived from grafted (**A**) fetus-NSPCs and (**B**) 2A4F iPSC-NSPCs. Graft survival rates of mice transplanted with (**C**) fetus-NSPCs and (**D**) 2A4F iPSC-NSPCs. Each of the same populations of fetus-NSPCs and 2A4F iPSC-NSPCs were transplanted into intact spinal cords (intact spinal cord group, black), injured spinal cords (injured spinal cord group, red), and injured spinal cords in the presence of FK506 (injured spinal cord + FK506 group, blue) in BALB/cA mice. Follow-up examinations were performed using bioimaging. Values represent the mean ± SEM. *P < 0.05, one-way ANOVA followed by the Tukey–Kramer test. The graft survival rates were assessed using Kaplan–Meier survival curves. *P < 0.05, Gehan-Breslow-Wilcoxon test. ns, not significant. In (**A**–**D**), N indicates the number of mice.

To assess the immunodynamics of transplanted cells in the injured spinal cord, we transplanted the same populations of fetus-NSPCs and 2A4F iPSC-NSPCs into intact spinal cords (intact spinal cord group), injured spinal cords (injured spinal cord group), and injured spinal cords with FK506 (injured spinal cord + FK506 group). Transplanted cell survival was assessed using bioimaging 3 months after transplantation (Fig. 6). We expected the luminescence to be lower in the injured spinal cord group than in the intact spinal cord group in the final follow-up examination, as the environment of the injured spinal cord was thought unlikely to be suitable for cell survival. However, we observed no significant difference in the survival of the transplanted cells in the intact spinal cord group and the injured spinal cord group following transplantation of both fetus-NSPCs (Fig. 6A and 2A4F iPSC-NSPCs (Fig. 6B). Our analysis of graft survival also revealed that the grafts were not rejected more frequently in the injured spinal cord than in the intact spinal cord. Remarkably, the survival of the fetus-NSPC grafts was higher in the injured spinal cord group, and the survival of iPSC-NSPC grafts was equivalent between groups (fetus-NSPCs grafts: 16.7% [n = 1/6] in the intact spinal cord group, 50.0% [n = 3/6] in the injured spinal cord group; 2A4F iPSC-NSPCs grafts: 33.3% [n = 2/6] in the intact spinal cord group, 33.3% [n = 2/6] in the injured spinal cord group) (Fig. 6C,D). Graft survival was 100% during the 8-week follow-up period in the injured spinal cord + FK506 group. Cessation of the FK506 treatment led to decreased luminescence after 1 week, and the transplanted fetus-NSPCs and 2A4F iPSC-NSPCs were ultimately rejected in 40% (n = 2/5) and 50% (n = 3/6) of the mice, respectively. During the subsequent 1-month follow-up, no mice exhibited new rejection of transplanted cells, and survival rates of fetus-NSPCs and 2A4F iPSC-NSPCs at 12 weeks after transplantation were 60% (n = 3/5) and 50% (n = 3/6), respectively. Notably, these rates are equivalent to those of the injured spinal cord group (Fig. 6C,D).

**Figure 7:**
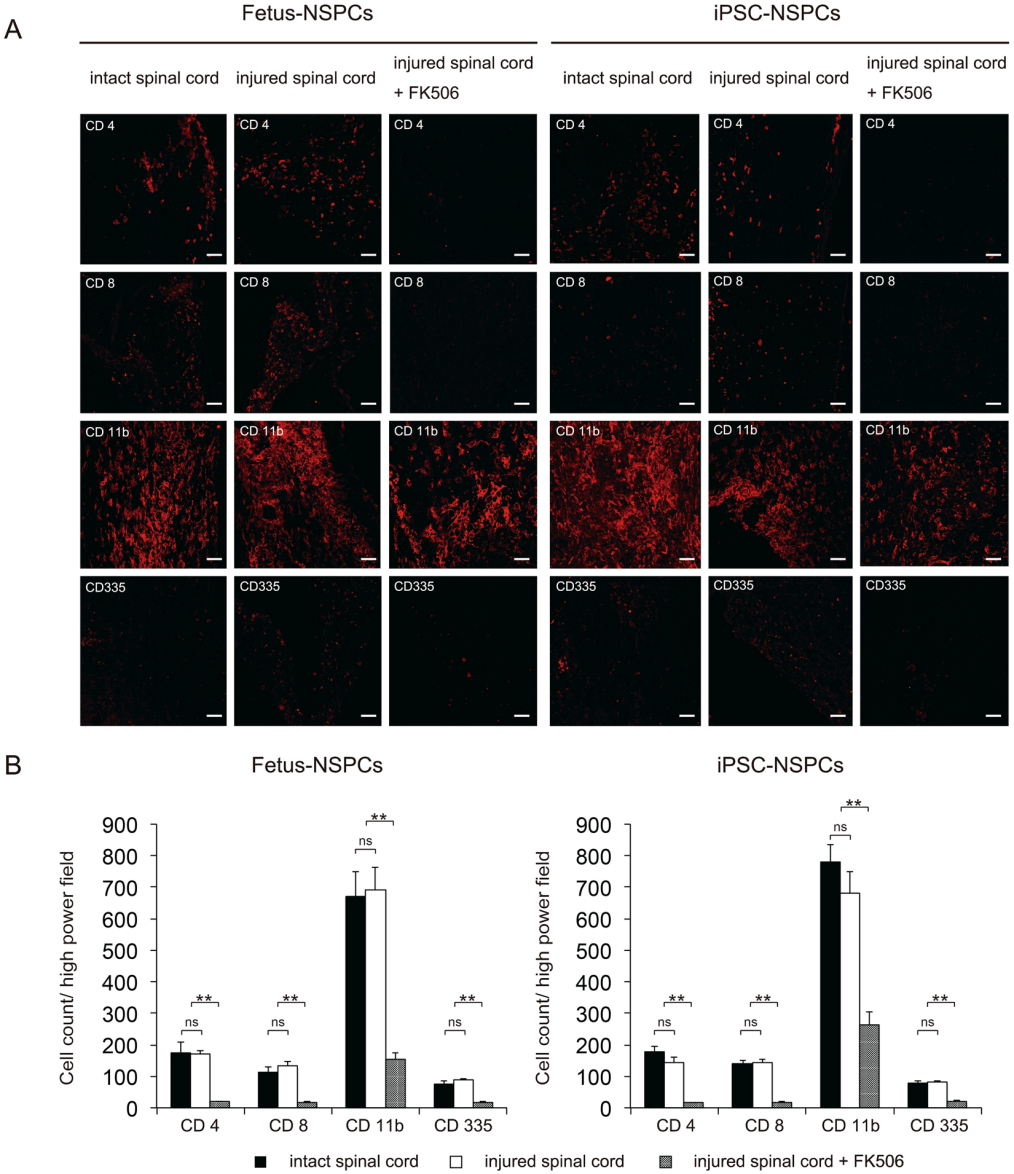
Histological examination of cells after transplantation into intact and injured spinal cords of BALB/cA mice. Fetus-NSPCs and 2A4F iPSC-NSPCs were transplanted into intact spinal cord, injured spinal cord, and injured spinal cord in the presence of FK506. Inflammatory cells (CD4, CD8, CD11b, and CD335) surrounding the surviving grafts were stained (**A**) and quantified (**B**). Values are shown as the mean ± SEM. *P < 0.005, unpaired two-tailed Student’s t-test and one-way ANOVA followed by the Tukey–Kramer test. ns, not significant. Scale bars in (**A**) = 100 µm.

We histologically examined fetus-NSPCs and 2A4F iPSC-NSPCs after allogeneic transplantation into intact and injured spinal cords. The degree of infiltration of CD4 + lymphocytes, CD8 + lymphocytes, CD11b + macrophages, and CD335 + NK cells was similar between groups transplanted with fetus-NSPCs and iPSC-NSPCs. No significant difference in the degree of inflammatory cell infiltration was observed between intact and injured spinal cords. In the injured spinal cord + FK506 group, the numbers of CD4 + cells, CD8 + cells, and CD335 + cells infiltrating the grafts were significantly reduced and barely detectable. The number of CD11b + macrophages was lowest in the group that received FK506, but many CD11b + macrophages remained in the grafts (Fig. 7A,B).

## Discussion

Questions surrounding immunogenicity and post-transplantation immunological dynamics of iPSC-derived products must be resolved before the transplantation of iPSC-derived cell products can be attempted^19^. In our study, we evaluated and compared the *in vitro* immunological characteristics of B6-derived fetus-NSPCs, which exhibit low immunogenicity^11^, with two lines of miPSC-NSPCs. We performed a series of transplantation experiments to evaluate the survival and dynamics of grafts in the spinal cord in syngeneic and allogeneic settings in mice, and compared the immunological tolerance of the transplantation site in intact and injured spinal cords.

Both fetus-NSPCs and iPSC-NSPCs exhibited minimal immunogenicity, which is consistent a previous examination of surface antigen expression in mouse ES cells^31^. The induction of differentiation increases the expression of MHC class I in mouse ES cells^32,33^. Studies of human ES cells and iPSCs showed that human leukocyte antigen (HLA) class I is expressed in undifferentiated ES cells and iPSCs and that its expression decreased after differentiation^34,35^. In the present study, MHC expression was barely detectable during differentiation from iPSCs to neurospheres (NS) at P1, P4, and P7 or following terminal differentiation into neurons, astrocytes, and oligodendrocytes. The expression of MHC class I and II and CD54 increased in the presence of IFNγ. In P7 NS cells from fetus-NSPCs and 2A4F iPSC-NSPCs, the expression levels of MHC class I and II were comparable to levels in blood cells; however, the levels remained very low in the other samples.

MLR assays showed that the lymphocyte activity tended to be higher in MHC-mismatched allogeneic settings than MHC-matched syngeneic settings, suggesting that the involvement of MHC class I and II is indispensable for lymphocyte activity. Fetus-NSPCs and iPSC-NSPCs showed a similar responsiveness to lymphocytes. Mesenchymal stem cells^36–38^ and NSPCs^39^ also exhibit similar immunosuppressive effects. An immunosuppressive effect on surrounding microglia mediated by TGFβ was found in NSPCs^13,40^ and ESCs^29^. The addition of a TGFβ-neutralizing antibody markedly increased CPM and SI in both fetus-NSPCs and iPSC-NSPCs, suggesting that iPSC-NSPCs exhibit TGFβ-dependent immunomodulatory activity.

The CPM was markedly higher in MLR assays using lymphocytes from mice that had previously rejected NSPCs or iPSC-NSPCs (immunized lymphocytes). A previous *in vivo* study using ES cells found that cells were rejected more rapidly after secondary transplantation^41^. Here, we showed increased activity for lymphocytes immunized with differentiation-induced fetus-NSPCs or iPSC-NSPCs, indicating that the memory of host lymphocytes was maintained and a secondary transplantation is more likely to induce an immune response. All 5 mice with previously rejected subcutaneous grafts, also rejected iPSC-NSPCs transplanted into the spinal cords (data not shown). These rejections may be attributable to immunological memory, such as the response of memory T cells^42,43^. Thus, in clinical practice, it may be necessary to use another cell type for a second transplantation if an initial graft is rejected.

The transplantation site can influence the immunogenicity of transplanted cells. Indeed, immune responses were induced to a greater extent in allogeneic transplantation settings when compared with syngeneic settings. To assess the effect of *in vitro* immunogenicity on post-transplantation immune rejection, we transplanted iPSC-NSPCs and fetus-NSPCs collected at P4 into the subcutaneous tissue of mice. The grafts survived for 3 months after syngeneic transplantation in all mice. In contrast, allogeneic subcutaneous transplantation of either fetus-NSPCs or iPSC-NSPCs resulted in immunological rejection and the disappearance of cells from all mice, which is consistent with previous studies. These results suggest that regimens involving the use of multiple immunosuppressive agents, such as those used for conventional organ transplantation, as well as the use of the closest available MHC-matched donor, may be required for transplantation into nonprivileged sites. Notably, after allogeneic transplantation into the intact spinal cord, both transplanted fetus-NSPCs and iPSC-NSPCs survived for 3 months in approximately 20% of the mice. Subsequently, the immune response persisted and grafts were rejected by some mice, whereas others established immune tolerance. Here the grafts survived for 1 year after transplantation, despite the absence of immunosuppressive agents. These results suggest that grafts are more likely to survive in immunologically privileged sites (e.g., the spinal cord) than grafts located in other nonprivileged sites (e.g., subcutaneous tissue).

Histological examination indicated greater inflammatory cell infiltration in the spinal cord after allogeneic transplantation when compared with syngeneic transplantation. The degree of infiltration was similar for transplanted fetus-NSPCs and iPSC-NSPCs. While CD11b + cells were the most common type of infiltrating cell, many CD163 + M2 macrophages were observed. M2 macrophages promote tissue repair, exert immunomodulatory effects^44^, and induce angiogenesis and produce immunosuppressive molecules, such as IL-10 and PGE2, in tumor tissues to suppress antitumor immunity^45,46^. In the present study, many infiltrating M2 cells were TGFβ +. A previous study demonstrated that mesenchymal stromal cells produce CC chemokine ligand 2 to recruit macrophages^47^. Similarly, NSPCs can recruit M2 macrophages at transplantation sites to promote survival while adjusting to the transplantation site environment. These behaviors are similar to those of cancer cells and tumor-associated macrophages from the peritumoral environment, which proliferate while interacting with the environment^48^.

Unlike the intact spinal cord, injured spinal cords have an inflammatory environment, often consisting of immunocompetent cells. Given the results obtained after the *in vitro* addition of IFNγ, we expected direct and indirect interactions with immune cells to increase the immunogenicity of cells transplanted into the injured spinal cord. The local environment, which exhibits alterations such as glial scarring after injury, was thought to be unsuitable for the survival of transplanted cells, and we expected graft rejection in a large number of mice. Surprisingly, however, in the present study in which the same populations of cells were transplanted following the same schedule, we found that graft survival tended to be higher in the injured spinal cord group than in the intact spinal cord group, although no significant differences were observed in photon counts after transplantation. These results suggest that the survival of transplanted cells in injured spinal cord may be at least equivalent to that in intact spinal cord. Histological examination also showed that the degree of inflammatory cell infiltration was equivalent between the intact and injured spinal cords. In the group receiving FK506, the graft infiltration of inflammatory cells, particularly CD4 and CD8 lymphocytes and NK cells, was reduced; this reduction may have contributed to the survival of transplanted cells.

Immune rejection can occur during organ transplantation even if multiple immunosuppressive agents are used. For example, transplanted iPSC-derived cardiomyocytes were rejected in non-human primates despite the use of three immunosuppressive agents (tacrolimus, mycophenolate mofetil, and prednisolone) in an MHC-mismatched setting, and transplanted cells in a partially MHC-matched setting did not survive without these three agents^25^. In our study, all grafts survived for a short follow-up period of 8 weeks in the presence of FK506; however, this result may be partly attributable to the allogeneic transplantation. Nevertheless, this finding suggests that in addition to the low *in vitro* immunogenicity and immunosuppressive effect of the transplanted graft, the spinal cord may be immunologically superior to other transplantation sites, and its environment may facilitate graft survival. Notably, graft survival decreased to the rate of the untreated group after the immunosuppressant treatment was discontinued. Thus, the regimen had no effect on the long-term survival of transplanted cells nor helped to establish immune tolerance. Future studies are needed to elucidate the mechanism of immune rejection and develop methods for establishing immune tolerance.

Previous studies estimated the post-allogeneic transplantation dynamics of human iPSC-NSPCs. In graft survival studies performed in xenogeneic settings, i.e., human cells (typically ES cells) transplanted into mice, the concomitant use of multiple immunosuppressive agents improved human cell survival^41,49^. Furthermore, the concomitant use of an anti-CD4 antibody and FK506 in our previous study promoted the survival of human iPSC-NSPCs in the injured spinal cords of mice^50^. Although the xenogeneic reaction of mouse immune cells to transplanted human cells was examined in these studies, the post-transplantation allogeneic reaction was not reproduced. Many recent studies attempted to reconstruct the human immune system *in vivo* in humanized mice, and the *in vivo* survival and function of transplanted human cells in humanized mice was reported^51,52^. In terms of the transplantation of immunocompetent cells, human T cells can now be differentiated *in vivo* in mice because these cells are matured in the mouse thymus so they do not induce human HLA-restricted immune responses. Therefore, all immune responses observed in humanized mice, including post-transplantation graft-versus-host disease, may be xenogeneic reactions, and human transplantation immunity has not been reproduced *in vivo* in mice. The *in vitro* evaluation of interactions between human lymphocytes and transplanted cells using MLR assays is useful for measuring the degree of rejection before transplantation, but only estimates are obtained because the degree of rejection varies according to the environment and transplantation site.

A limitation of this study is that it was conducted in allogeneic and syngeneic mouse systems, which may differ considerably from the human immune system. However, it is currently impossible to completely reproduce the immunodynamics of iPSC-derived products after transplantation into humans. The results of our study suggest that fetus-NSPCs and iPSC-NSPCs exhibit similar immunogenicity. In addition to the post-transplantation dynamics of human fetus-NSPCs, the comparison of the immunogenicity of human fetus-NSPCs and human iPSC-NSPCs may provide a basis for estimating the immunodynamics of human iPSC-NSPCs following transplantation into humans. The results of the present study also suggest that therapeutic cell transplantation into the spinal cord may be more immunologically tolerated than transplantation into other organs, which may be advantageous for clinical applications.

Finally, it will be also important to compare the therapeutic potential of fetus-NSPCs and iPSC-NSPCs, which will be addressed in our future study by evaluating the functional recovery after grafting these cells into spinal cord injury model mice.

## Methods

### Study approval

All experiments were performed in accordance with the Guidelines for the Care and Use of Laboratory Animals of Keio University (Assurance No. 13020) and the Guide for the Care and Use of Laboratory Animals (National Institutes of Health, Bethesda, MD, USA).

### Fetus-NSPCs, iPSCs, iPSC-NSPCs culture, neural induction and lentiviral ffLuc transduction

Cell culturing of fetus-NSPCs^26^ and miPSC-NSPCs (clones 2A3F and 2A4F^24^) as well as neural induction were performed as previously described^4,53–55^, with minor modifications. For the fetus-NSPCs cultures, the striata of transgenic mice ubiquitously expressing ffLuc-cp156 (a fusion protein of firefly luciferase and circularly permuted Venus protein^26^), were dissociated using a fire-polished glass pipette on embryonic day 14. The dissociated cells were collected by centrifugation and resuspended at a density of 1 × 10^6^ cells/mL in media hormone mix (MHM) supplemented with B27 and 20 ng/mL of FGF-2 (Peprotech, Rocky Hill, NJ, USA) for 7 days. For the iPSC-NSPCs, iPSCs were grown on gelatin-coated (0.1%) culture dishes; irradiated murine embryonic fibroblasts (MEFs), maintained in standard ES cell medium, were used for embryoid body (EB) formation, as described previously^4,56^. Retinoic acid (10^−8^ M; Sigma Chemical Co., St. Louis, MO, USA) was added on the day 2. Seven days after EB formation, the EBs were enzymatically dissociated with TrypLE Select into single cells and cultured in suspension in the same media hormone mix (MHM) medium for seven days to allow neurosphere formation. For neurosphere passages, the fetus-NSPCs and iPSC-NSPCs were dissociated using TrypLE Select and then cultured in medium every seven days. The primary, 4^th^, and 7^th^ neurospheres were used for *in vitro* experiments. Some cells were treated with 25 ng/mL of recombinant murine interferon-γ(IFNγ; Peprotech) for 48 hours prior to evaluation, and 10^−6^ M of SB431542 (Sigma Chemical Co.), which is an inhibitor of transforming growth factor β(TGFβ) superfamily type I activin receptor-like kinase (ALK) receptors. The ffLuc lentivirus was prepared and transduced into iPSC-NSPCs according to a method described previously^50^. Briefly, the 3^rd^ neurospheres were dissociated and transduced with lentivirus-expressing ffLuc (Venus fluorescent protein fused to firefly luciferase)^26^ under the control of an elongation factor promoter (pCS II-EF-dVenus-Luc2). The resulting neurospheres were then passaged to the 4^th^ neurospheres and used for transplantation.

### Flow cytometric analysis of expression of immune related surface markers

Fetus-NSPCs, iPSCs, P1, P4, P7 iPSC-NSPCs and isolated peripheral blood leukocytes were analyzed using immunofluorescence staining in the presence or absence of IFNγ, followed by flow cytometry. Before the evaluation of the iPSCs, detached iPSCs were cultured on gelatin-coated (0.1%) culture dishes for two hours, to eliminate the MEFs. Fetus-NSPCs and iPSC-NSPCs were dissociated using Accutase (BD Biosciences, Franklin Lakes, NJ, USA) following the manufacturer’s instructions. BALB/cA mouse spleen cells were treated with 10 **×** RBC lysis buffer (eBioscience Inc., San Diego, CA, USA) according to the manufacturer’s instructions. The cells were stained with the following primary antibodies: fluorescein isothiocyanate (FITC)-labeled anti-mouse H-2 (clone M1/42; BioLegend Inc., San Diego, CA, USA), phycoerythrin (PE)-labeled anti-mouse H-2 (clone M1/42; BioLegend Inc.), allophycocyanin (APC)-labeled anti-mouse I-A/I-E (clone M5/114.15.2; BioLegend Inc.), APC-labeled anti mouse intercellular adhesion molecule-1 (ICAM-1)/CD 54 (R&D Systems Inc., Minneapolis, MN, USA), FITC-labeled anti-mouse CD 40 (clone 3/23; BioLegend Inc.), PE/Cy7-labeled anti-mouse CD 40 (clone 3/23; BioLegend Inc.), APC-labeled anti-mouse CD 80 (clone 16-10A1; BioLegend Inc.), PE-labeled anti-mouse CD 86 (clone GL-1; BioLegend Inc.), PE-labeled anti-mouse CD 152 (clone UC10-4B9; eBioscience Inc.), PE-labeled anti mouse Rae-1(pan-specific) (R&D Systems Inc.), Rat IgG2a K Isotype Control FITC (clone RTK2758; BioLegend Inc.), Armenian Hamster IgG Isotype Control APC (clone HTK888; BioLegend Inc.), Rat IgG2b K Isotype Control APC (RTK4530; BioLegend Inc.), Rat IgG2a K Isotype Control PE (RTK2758; BioLegend Inc.), Rat IgG2a K Isotype Control PE/Cy7 (RTK2758; BioLegend Inc.), Rat IgG2b K Isotype Control FITC (eB149/10H5; eBioscience Inc.) and Armenian Hamster IgG Isotype Control PE (eBioscience Inc.). The cells were stained with a mixture of the primary antibodies at 4 °C for 30 min. Flow cytometry was performed using a fluorescence activated cell sorting (FACS) Verse^TM^ instrument (BD Biosciences) and FlowJo software (Tree Star, Ashland, OR, USA).

### Mixed lymphocyte reaction (MLR)

MLR experiments were performed by co-culturing single-cell suspensions of mouse PBMCs from the spleens of C57BL6/J and BALB/cA mice with irradiated fetus-NSPCs and iPSC-NSPCs in a U-bottom 96-well plate. Briefly, PBMCs from the spleens of C57BL6/J mice (syngeneic), BALB/cA mice (allogeneic), and BALB/cA mice that had undergone a spinal cord transplantation and rejection of the NSPCs and iPSC-NSPCs (allogeneic, immunized) were prepared by centrifuging peripheral blood using Lymphoprep (Axis-Shield PoC AS, Oslo, Norway) according to the manufacturer’s instructions. A cell mixture containing 1 × 10^4^ cells from irradiated (20 Gy) fetus-NSPCs and iPSC-NSPCs (9–12 days after passage) and 1 × 10^5^ cells from PBMCs were maintained in 200 μL of Roswell Park Memorial Institute (RPMI)-1640 medium (Sigma Chemical Co.) with or without SB431542 in 96-well plates at 37 °C under 5% CO_2_. Five days after the start of the mixed culture, 1 μCi of^3^H-thymidine was added. Using a radioactivity counter, the amount of^3^H-thymidine incorporated into the cells after 24 h was measured and then reported as the CPM value. The SI was calculated by dividing the CPM for the allogeneic MLR by the CPM for the syngeneic MLR.

### Spinal cord injury (SCI) model and transplantation of iPSC-NSPCs

Female 8-week-old C57BL6/J mice and BALB/cA mice (20–22 g) were anesthetized with an intraperitoneal injection of ketamine (100 mg/kg) and xylazine (10 mg/kg). After laminectomy at the 10^th^ thoracic spinal vertebra, the dorsal surface of the dura mater was exposed. Contusive spinal cord injury was induced at the Th10 level using an infinite horizon impactor (60 kdyn; Precision Systems and Instrumentation, Lexington, KY, USA). Nine days after injury, 5 × 10^5^ iPSC-NSPCs were transplanted into the lesion epicenter using a glass micropipette at a rate of 1 µL/min with a 25-µL Hamilton syringe and stereotaxic microinjector (KDS 310, Muromachi Kikai Co., Ltd, Tokyo, Japan). All surgeries were performed under anesthesia, and all efforts were made to minimize animal suffering.

### Bioluminescence imaging

A Xenogen IVIS Spectrum *in vivo* imaging system (Caliper Life-Sciences, Hopkinton, MA, USA) was used for the bioluminescence imaging of surviving transplanted iPSC-NSPCs. Imaging was performed once each week after cell transplantation. Briefly, D-luciferin (Promega, Madison, WI, USA) was administered via intraperitoneal injection at a dose of 300 mg/kg body weight. The mice were placed in a light-tight chamber and photons emitted from the luciferase-expressing cells were collected at integration times of 5 s to 2 min, depending on the intensity of the bioluminescence emission. The bioluminescence signals were quantified as maximum radiance units (photons per second per centimeter squared per steradian [p/s/cm^2^/sr]) and represented as the log10 values (photons per second).

### Histology

Mice were anesthetized and transcardially perfused with 0.1 M phosphate-buffered saline containing 4% paraformaldehyde. The spinal cords were removed, embedded in Optimal Cutting Temperature compound (Sakura Finetechnical Co., Ltd., Tokyo, Japan), and sagittaly sectioned on a cryostat (Leica CM3050 S; Leica Microsystems, Buffalo Grove, IL, USA). Sections were stained with hematoxylin-eosin (HE), Hoechst 33258 dye (10 µg/mL; Sigma Chemical Co.), and the following primary antibodies: anti-green fluorescent protein (GFP) (rabbit IgG, 1:200; Frontier Institute Co., Ltd., Hokkaido, Japan), anti-β-tubulin isotype III (mouse IgG, 1:1,000; Sigma Chemical Co.), anti-glial fibrillary acidic protein (anti-GFAP, rabbit IgG, 1:200; Dako, Carpinteria, CA, USA), anti-CNPase (mouse IgG, 1:1000; Sigma Chemical Co.), anti-adenomatous polyposis coli CC-1 (APC CC-1, mouse IgG, 1:200; Abcam, Cambridge, UK, ab16794), anti-MHC class I (clone ER-HR 52, mouse IgG2a, 1:100; Abcam, Cambridge, UK), anti-MHC class II (clone MRC OX-6, Rat IgG1, 1:100; Abcam), anti-CD4 (clone RM4-5, Rat IgG2a K, 1:500; BD Pharmingen^TM^, San Diego, CA, USA), anti-CD8a (clone 53-6.7, mouse, 1:200; eBioscience), anti-CD11b (rat IgG, 1:200; BD Pharmingen^TM^), anti-CD335 (NKp46, clone 29A1.4, Rat IgG2a K, 1:200; BioLegend Inc.), anti-CD163/M130 (Rabbit IgG, 1:200; Bioss Inc., Wobum, MA, USA) and anti-TGFβ (Rabbit IgG, 1:200; Abcam). To quantify the CD4-, CD8-, CD11b- and CD335-positive cells, three representative mid-sagittal sections were selected, and five regions within 1 mm rostral and caudal to the lesion epicenter were automatically captured at 200× magnification. The numbers of marker-positive cells were counted for each section (n = 1 for the fetus-NSPCs transplanted into intact spinal cord group, n = 2 for each of remaining group).

### Graft survival analysis

Graft rejection was defined according to the following criteria: 1) a bioluminescence photon level that declined to the background level with no subsequent increase and 2) a histological analysis did not reveal transplanted cells in the transplanted site (five slices/mouse). Graft survival rate was calculated by dividing the number of non-rejected mice by the total number of mice in each group

### Statistical analysis

All data are represented as the mean ± standard error of the mean (SEM). Differences in the MLR assay and quantitative analysis of CD4+, CD8+, CD11b+, and CD335+ cells were analyzed using an unpaired two-tailed Student’s t-test or one-way analysis of variance (ANOVA) followed by the Tukey–Kramer test. Differences in surface protein expressions and CPM were analyzed using a one-way ANOVA followed by the Tukey–Kramer test. The graft survival rates were analyzed using the Gehan-Breslow-Wilcoxon test. All statistical analyses were performed using Prism (GraphPad Software). The significance levels were set at *P < 0.05 and **P < 0.005 for all statistical analyses.

## Electronic supplementary material

supplemental figures
