# Supplementary material for: Low immunogenicity of mouse induced pluripotent stem cell-derived neural stem/progenitor cells
Source: Sci Rep. 2017 Oct 11;7:12996. doi: 10.1038/s41598-017-13522-w (PMC5636829; doi:10.1038/s41598-017-13522-w)
Supplement: Supplementary file 1 — supplemental figures [file 41598_2017_13522_MOESM1_ESM.pdf]

Low immunogenicity of mouse induced pluripotent stem cell-derived neural stem/progenitor cells

Go Itakura[1,2], Masahiro Ozaki[1,2], Narihito Nagoshi[2], Soya Kawabata[1,2], Yuichiro Nishiyama[1,2], Keiko Sugai[1,2], Tsuyoshi Iida[1,2], Rei Kashiwagi[1,2], Toshiki Ookubo[1,2], Kaori Yastake[1,2], Kohei Matsubayashi[1,2], Jun Kohyama[1], Akio Iwanami[2], Morio Matsumoto[2], Masaya Nakamura[2*] and Hideyuki Okano[1*]

[1]Department of Physiology, Keio University School of Medicine, 35 Shinanomachi, Shinjuku, Tokyo 160-8582, Japan

[2]Department of Orthopaedic Surgery, Keio University School of Medicine, 35 Shinanomachi, Shinjuku, Tokyo 160-8582, Japan

*Corresponding authors

Contact information: hidokano@a2.keio.jp (H.O.) and masa@a8.keio.jp (M.N.)

Running title: Low immunogenicity of mouse iPSC-NSPCs

Key words: induced pluripotent stem cells (iPSCs), neural stem/progenitor cells (NSPCs), immune-rejection, syngenic, allogenic.

Supplementary figures

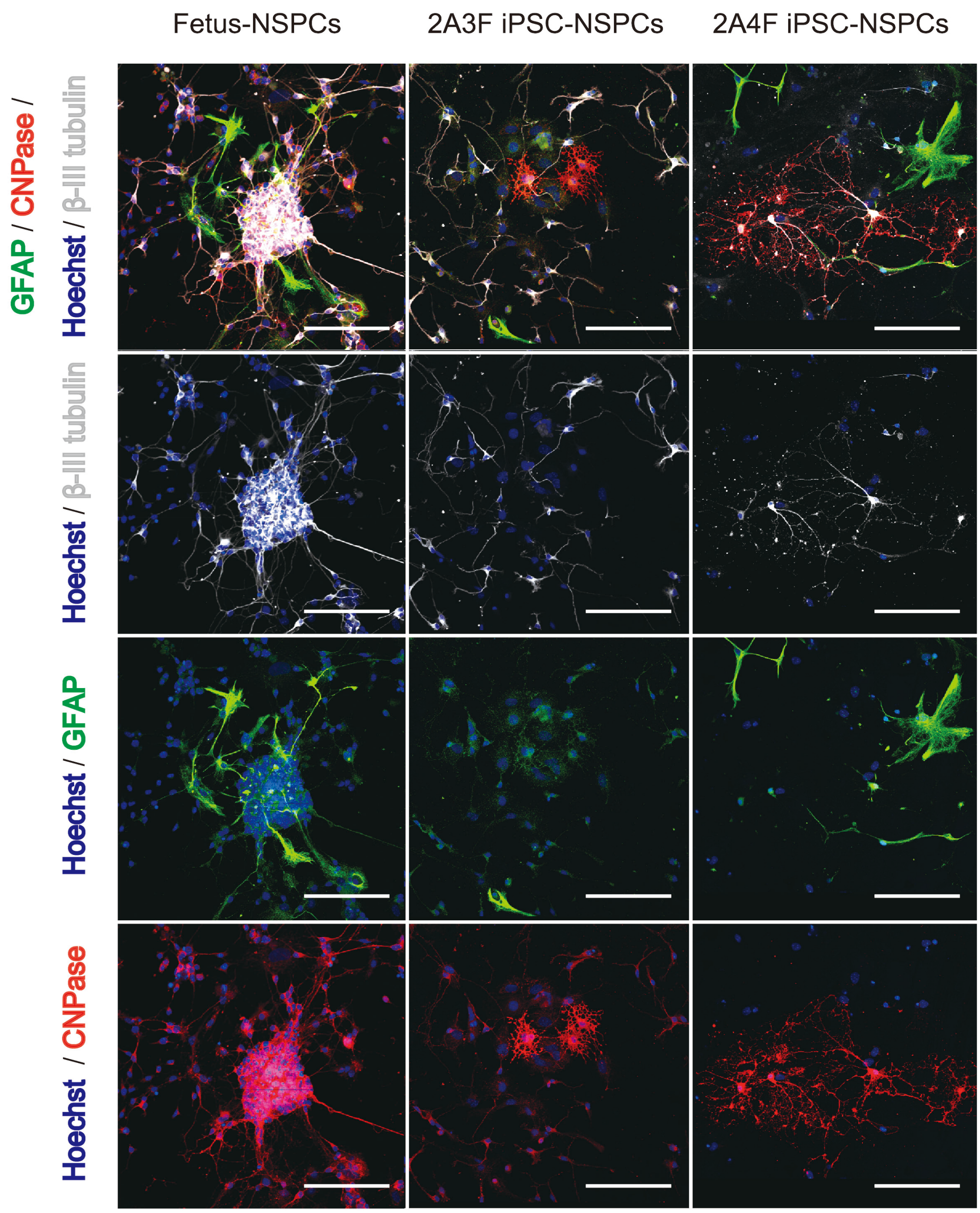

Figure S1. High magnification images of terminally differentiated NSPCs in vitro.

Terminally differentiated neurons, astrocytes, oligodendrocytes from fetus-iPSC-NSPCs

and iPSC-NSPCs were stained.

iPSC-NSPCs differentiated into neurons, astrocytes and oligodendrocytes similar to the fetus-NSPCs. Scale bars = 100 μm.

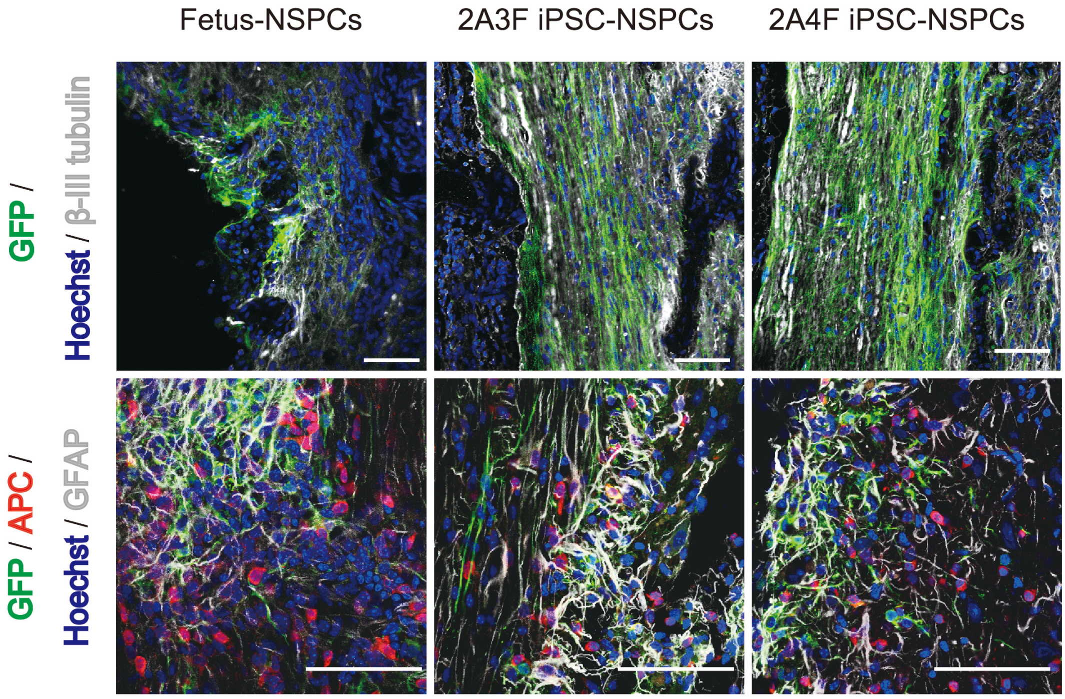

Figure S2. High magnification images of terminally differentiated NSPCs in vivo.

Fetus-NSPCs and iPSC-NSPCs differentiated into neurons, astrocytes and oligodendrocytes, and integrated with host cells. Scale bars = 100 μm.
